# Evolution of DNA Replication Protein Complexes in Eukaryotes and Archaea

**DOI:** 10.1371/journal.pone.0010866

**Published:** 2010-06-02

**Authors:** Nicholas Chia, Isaac Cann, Gary J. Olsen

**Affiliations:** 1 Institute for Genomic Biology, University of Illinois at Urbana-Champaign, Urbana, Illinois, United States of America; 2 Loomis Laboratory of Physics and the Physics Frontier Center: Physics of the Living Cell, University of Illinois at Urbana-Champaign, Urbana, Illinois, United States of America; 3 Department of Microbiology, University of Illinois at Urbana-Champaign, Urbana, Illinois, United States of America; 4 Laboratory of Animal Sciences, University of Illinois at Urbana-Champaign, Urbana, Illinois, United States of America; University of Hyderabad, India

## Abstract

**Background:**

The replication of DNA in Archaea and eukaryotes requires several ancillary complexes, including proliferating cell nuclear antigen (PCNA), replication factor C (RFC), and the minichromosome maintenance (MCM) complex. Bacterial DNA replication utilizes comparable proteins, but these are distantly related phylogenetically to their archaeal and eukaryotic counterparts at best.

**Methodology/Principal Findings:**

While the structures of each of the complexes do not differ significantly between the archaeal and eukaryotic versions thereof, the evolutionary dynamic in the two cases does. The number of subunits in each complex is constant across all taxa. However, they vary subtly with regard to composition. In some taxa the subunits are all identical in sequence, while in others some are homologous rather than identical. In the case of eukaryotes, there is no phylogenetic variation in the makeup of each complex—all appear to derive from a common eukaryotic ancestor. This is not the case in Archaea, where the relationship between the subunits within each complex varies taxon-to-taxon. We have performed a detailed phylogenetic analysis of these relationships in order to better understand the gene duplications and divergences that gave rise to the homologous subunits in Archaea.

**Conclusion/Significance:**

This domain level difference in evolution suggests that different forces have driven the evolution of DNA replication proteins in each of these two domains. In addition, the phylogenies of all three gene families support the distinctiveness of the proposed archaeal phylum Thaumarchaeota.

## Introduction

DNA replication is one of the defining processes of modern life. The spread of DNA replication likely represents a major evolutionary transition in early life. Duplication of DNA content allows organisms to pass genetic information onto future generations. Mutations during the duplication process enable populations to evolve and adapt. The centrality of DNA replication to such important life processes makes the evolution of the DNA replication machinery all the more significant for understanding the evolution of life.

Chromosome replication in Archaea and eukaryotes requires three ancillary complexes—the proliferating cell nuclear antigen (PCNA), replication factor C (RFC), and the minichromosome maintenance complex (MCM) [1]–[3]. Each of these three complexes plays an essential role in DNA replication. The MCM complex is thought to function as replicative DNA helicases that unwind the DNA at the replication fork, and PCNA and RFC, known as the clamp and clamp loader, respectively, confer the processive DNA synthesis to the DNA polymerase [1]–[3]. Without them, large genomes would be extremely difficult to sustain.

We refer the interested reader to Refs. [1]–[3] for more in-depth reviews of the proteins that act at the replication fork; here we provide only an outline sufficient to introduce the three complexes that we analyze. The process of DNA replication generally begins at specific sites known as origins of replication. The double-stranded DNA is unwound and the two single strands form the templates for replication of the chromosome. The site of DNA replication activity is known as the replication fork, and the supramolecular assembly carrying out the process of replication is known as the replisome. The replisome consists of a large number of protein complexes. Replicative DNA polymerases are incapable of *de novo* DNA synthesis. Therefore, once the single stranded DNA template is generated by the replicative helicase, an RNA primer is initially synthesized by a DNA primase to create a primer/template junction. The primer/template junction is recognized by the clamp loader, which loads the clamp onto this DNA structure. The clamp then recruits the DNA polymerase to the single stranded DNA to perform the actual template guided process of DNA replication. The function of PCNA is to encircle the DNA and affix, or clamp, the polymerase to the template. In a role analogous to the bacterial beta clamp, PCNA enhances the speed and efficiency of DNA polymerase by enabling the polymerase to synthesize the complementary strand continuously without frequent dissociation.

**Figure 1:**
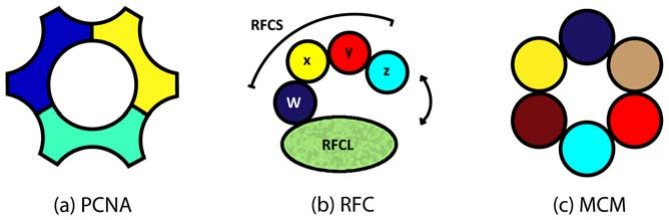
Structural schematic of the PCNA, RFC, and MCM complexes. (a) PCNA consists of 3 subunits forming a ring-like clamp that encloses the DNA polymerase and single stranded DNA. (b) RFC consists of a total of five subunits. Four small subunits (RFCS) form a chain, whose positions are labeled 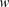, 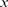, 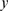, and 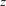, that is anchored by 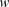 RFCS to one large subunit (RFCL). The complex opens between the terminal 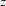 RFCS and RFCL via an ATP driven conformation change. (c) The MCM complex consists of six MCM proteins in a hexameric ring.

Figure 1 shows the general subunit organization of PCNA, RFC, and MCM in the archaeal and eukaryotic domains [3], [4]. A common theme of these complexes is the repetitive use of homologous or identical subunits. For instance, although PCNA is always a trimer, with the three subunits in a ring (Fig. 1a), the subunits can be of 1, 2, or 3 different sequence types corresponding to 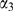, 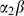, and 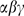 subunit compositions. In eukaryotes, the subunits are all identical, forming a homotrimer, but among the Archaea there is a greater diversity. In the case of RFC, there is always the distinct large subunit (RFCL), while the smaller subunits (RFCS) are of 1,2, or 4 different sequence types. In the case of MCM helicase, the six subunits are drawn from 1, 2, 3, 4, 6, or 8 distinct sequence types, depending on the phylogenetic group. The diversity of sequence types is summarized by phylogeny in Table 1.

**Table 1:** Number of PCNA, RFCS, and MCM subunits found in Archaea and eukaryotes for literature [1], [3], [21]–[23], [27], [28], [33], [51], [52], [59], [66], [67] and this work.

	Number of distinct subunits		
Taxonomic Unit	PCNA	RFCS	MCM
Archaea			
Crenarchaeota	1,2,3	1,2	1
Euryarchaeota	1,2	1,2	1–4,8
Korarchaeota	1	1	1
Nanoarchaeota	1	1	1
eukaryotes	1	4	6
total number of subunits in structure	3	4	6

In all cases where distinct sequence types are observed within a complex, the proteins are sufficiently similar to imply a common ancestry. For over 40 years it has been observed that gene duplication followed by divergence is an important source of new or modified protein functions [5], [6]. The globins are one of the earliest elucidated examples of a protein family that arose from gene duplications [7], [8]. Gene family expansions are often associated with the emergence of organismal complexity [5], [9]. The number of examples linking increasing organismal complexity and gene duplication continues to grow [10], [11]. In fact, the *Saccharomyces cerevisiae* genome appears to be the result of the duplication of a smaller ancestral genome [12]. Such genome duplications have been postulated to be key steps in the increasing complexity of microbes [13] and vertebrates [5].

The extensive role and implications of gene duplication in the evolution for increasing complexity speak to a larger puzzle. The question of emergence of complexity [14], [15] encompasses everything from the emergence of early life chemistry [16], [17] to higher eukaryotes [5], [18] and everything in between [13], [19]. In this work, we examine parallel questions about the role of gene duplication and divergence in shaping complexity. The complexity we examine arises from within each of the three protein complexes, and the source of this complexity can be traced by uncovering the evolutionary relationships between the various subunits.

Complexes consisting only of repeated identical subunits are simpler than complexes consisting entirely of homologous, but not identical, subunits. As such, the number of distinct sequence types in each complex serves as a proxy for the overall level of complexity. We trace the emergence of the distinct sequence types in order to put together a picture of how such complexity arose. For instance, where did the distinct subunits come from? Were more specialized subunits invented once and subsequently horizontally gene transferred (HGT) or did complexity increase independently in different lineages? Did simpler complexes with less specialized subunits beget the more specialized subunits in the complexes consisting of distinct subunits, or vice-versa?

## Results

With these questions in mind, we examine the phylogeny of the PCNA, RFCS, and MCM subunits. The phylogenetic data is then compared in detail with the known biochemistry of each subunit, in particular, a subunits interaction partners within each complex.

### Proliferating Cell Nuclear Antigen

PCNA was so named after it was found to be highly abundant in proliferating cells [20]. PCNA consists of three subunits (Figure 1a) of 1, 2, or 3 sequence types, depending on the phylogenetic group (Table 1). In the interest of clarity and consistency, we introduce our own designations of the PCNA subunits (C1, C2, C3). Table 2 translates our notation to that of previous literature [21]–[23].

**Table 2:** Crenarchaeotal PCNA nomenclature.

Organism	PCNA C1	PCNA C2	PCNA C3	Reference
*Aeropyrum pernix*	*Ape*PCNA2	*Ape*PCNA3	*Ape*PCNA1	[22]
*Sulfolobus solfataricus*	*Sso*PCNA2	*Sso*PCNA1	*Sso*PCNA3	[21]
*Sulfolobus tokodaii*	*Sto*PCNA3	*Sto*PCNA2	*Sto*PCNA1	[23]

**Figure 2:**
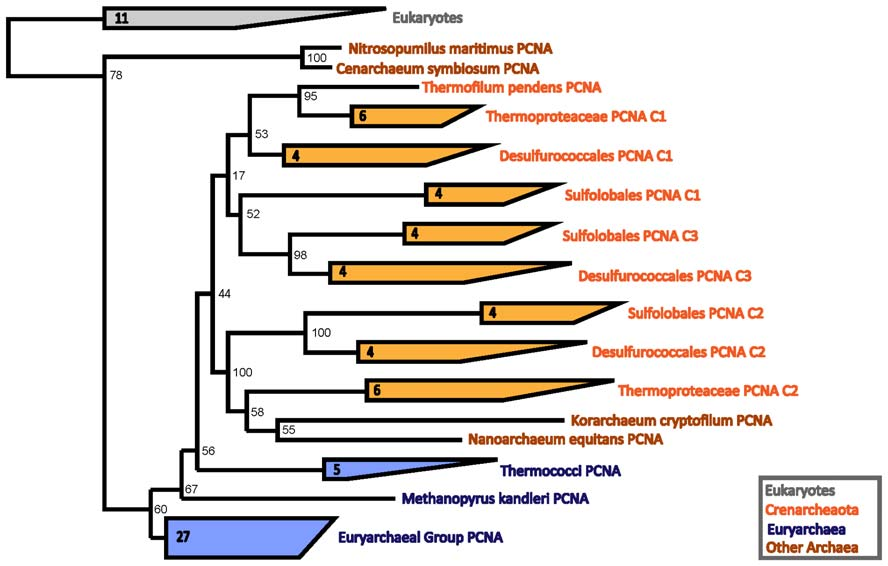
PCNA phylogeny, rooted between the Archaea and the eukaryotes. Tree produced using RAxML [63]. Note the proliferation of distinct subunit types in the Crenarchaeota.

The maximum likelihood phylogeny of the PCNA subunits is shown in Figure 2. This resultant phylogeny generally agrees with the NCBI taxonomy of the corresponding organisms. For clarity, more closely related sequences are shown as a collapsed group. The archaeal and eukaryotic sequences are grouped into separate clades. The Crenarcheota and the Euryarchaea also form distinct groups. The placement of *Nitrosopumilis* and *Cenarcheaum* in Figure 2 is consistent with recent proposals that these organisms belong to a phylum distict from the Crenarchaeota and Euryarchaea, which has been named Thaumarchaeota [24]. The *Korarchaeum* and *Nanoarchaeum* sequences are grouped together within those of the Crenarchaeota. Given the general agreement between the PCNA phylogeny and the organismal taxonomy, HGT does not appear to have occurred.

The eukaryotes and the Euryarchaeota contain only one PCNA gene, with the exception of a few near identical copies of unknown functionality in *Drosphila*, *Arabidopsis*, and *Thermococcus* (see Figure S1) that are generally not present in closely related taxa (data not shown). By contrast, the Crenarchaeota show deep branchings between PCNA subunits. *Cenarchaeum symbiosum* contains one PCNA gene, while the Thermoproteales have either one, as in *Thermofilum pendens*, or two distinct PCNA encoding genes, as in the Thermoprotaeceae. The Desulfurococcales and the Sulfolobales both encode three distinct PCNA subunits.

**Figure 3:**
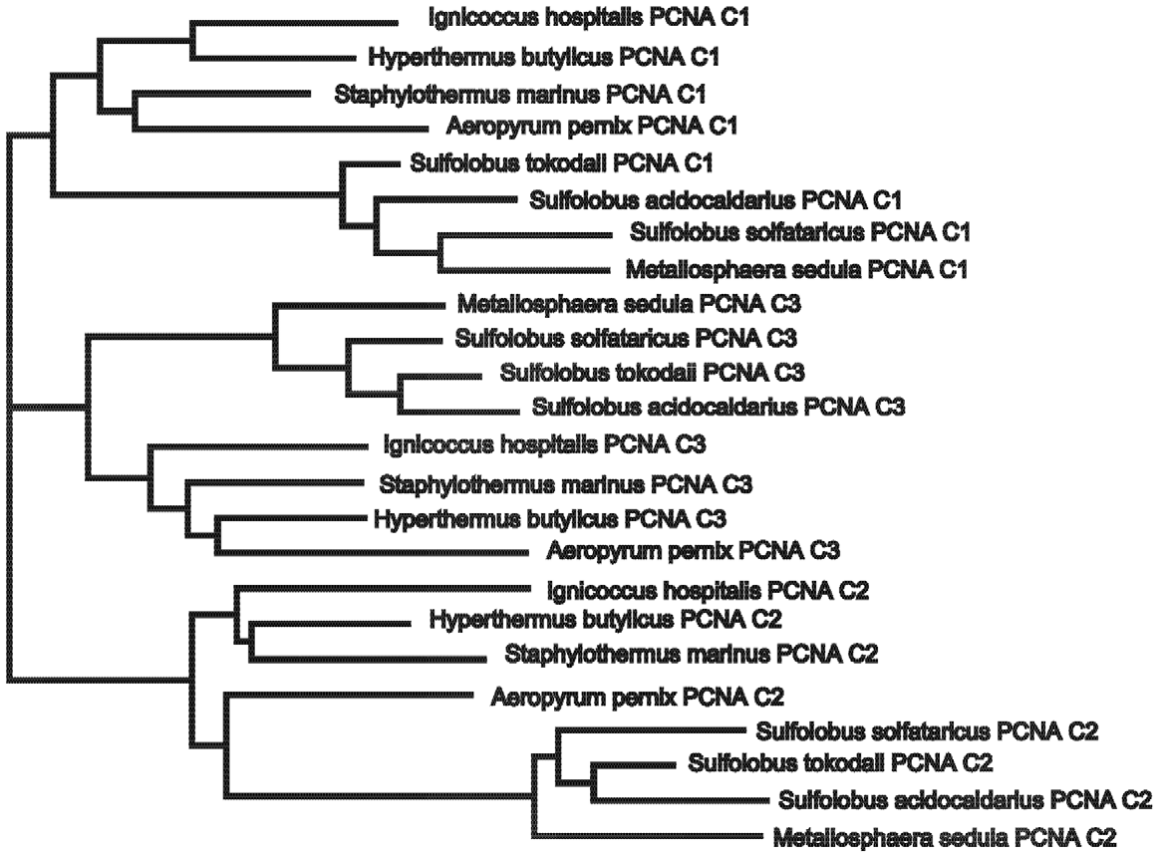
Desulfurococcales and Sulfolobales PCNA phylogeny rooted between PCNA C1, C2, and C3. The branching indicated here lends further support to the three PCNA C1, C2, and C3 groupings.

The phylogenetic relationships between the distinct sequence types yield an interesting picture—one that is consistent with their known biochemical properties. Note that the three distinct types of PCNA roughly group into three clades labeled C1, C2, and C3. Sulfolobales PCNA C1 appears slightly more related to PCNA C3, but not significantly so. We tested this further by constructing a phylogeny of sequences from organisms with more than one distinct sequence type. As shown in Figure 3, in this more focused phylogeny, the PCNA subunits C1, C2, and C3 all group separately.

Furthermore, within each of these three groups, the subunits share similar interaction properties. PCNA C1 appears to have preserved the most ancestral function, sharing the most properties in common with the homotrimeric PCNA subunit. C1 has the most stable dimeric interactions with the other subunits [21]–[23] and in *Aeropyrum pernix*, C1 is capable of forming a homotrimer [22]. In addition, C1 is present in all heterotrimeric configurations of PCNA (C1-C2-C3, C1-C1-C2, and C1-C2-C2) [21]–[23]. Phylogenetically, C1 is also the most closely related to the homotrimeric PCNA of *Thermofilum pendens* (Figure 2).

In contrast, C3 takes part only in C1-C2-C3 heterotrimer arrangements [21]–[23]. Data suggest that in *Sulfolobus solfataricus*, C3 is the last to be recruited into the PCNA trimer [21]. Overall, C3 has the least interactions with the other subunits [21]–[23] and appears to be the most functionally divergent of the three subunits from homotrimeric PCNA.

The results for PCNA are consistent with a simpler ancestral homotrimeric PCNA subunit and subsequent duplication and divergence of the distinct subunit types. The archaeal and eukaryotic PCNA both appear to have diverged from a homotrimeric form. Then, in the crenarcheaotes, more specialized PCNA sequence types appear to have originated from gene duplications, while the eukaryotes and Euryarchaea retained the ancestral configuration.

### The Clamp Loader: Replication Factor C

The RFC complex consists of five subunits, one large (RFCL) and four small (RFCS). The RFC complex opens between the 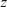-position RFCS and the RFCL (Figure 1b) in order to open and close PCNA about the DNA polymerase at the replication fork [25], [26]. The RFC complex is made up of either 1, 2, or 4 distinct RFCS sequence types, depending on phylogenetic group (Table 1).

**Figure 4:**
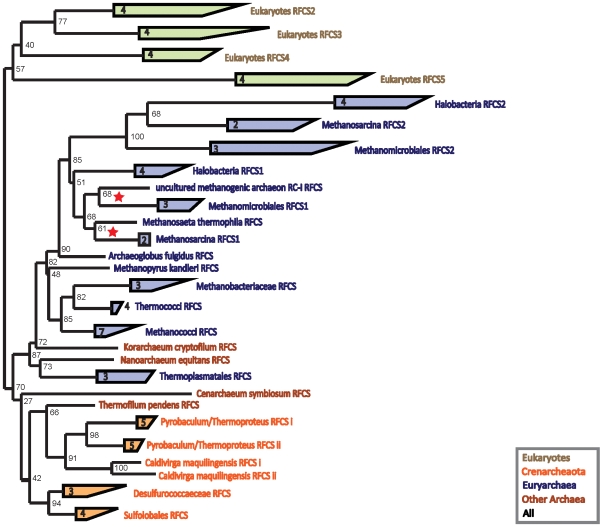
RFCS subunit phylogeny rooted between the Archaea and the eukaryotes. The red stars indicate splits between RFCS and RFCS1 subunit types in the Methanomicrobia, possibly from loss of RFCS2.

The maximum likelihood phylogeny of the RFCS subunits is shown in Figure 4. Again, the phylogeny shows general agreement with the NCBI taxonomy of the corresponding organisms. As such, HGT does not appear in the phylogeny of the RFCS subunits. The eukaryotes, crenarchaeotes, and Euryarchaea form separate groups. As with PCNA, the RFCS tree places the *Cenarcheaum* deep in the branching of archaeal sequences, again consistent with proposals that it be a member of a distinct phylum. The Korarchaea and Nanoarchaea sequences cluster with those of the Euryarchaea. The rooting between the eukaryotes and Archaea follows the canonical pattern, dividing the crenarchaeotes and the Euryarchaea at the base of the archaeal clade.

The phylogeny of the RFCS subunits shows that a RFC with four distinct RFCS sequence types seems to have been present in a common eukaryotic ancestor. This can be seen from the four eukaryotic RFCS clades—one for each RFCS position. On the other hand, the archaeal RFC consists of one or two distinct RFCS subunits [27], [28]. Archaea containing only one distinct RFCS form the RFC complex with the same RFCS in all four positions [25]. Euryarchaeal RFC complexes with two distinct RFCS subunits are composed of three RFCS1 at positions 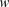, 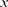, and 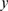, and a single RFCS2 at position 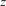 [29]. The configuration of RFC in crenarchaeotes with two distinct subunits has not yet been elucidated.

In Euryarchaeota, the specialization of RFCS into RFCS1 and RFCS2 appears to have occurred before the split between Methanomicrobia and Halobacteria. Following the RFCS1-RFCS2 divergence, there appear to be two independent losses of RFCS2 in the Methanomicrobia, indicated by stars in Figure 4. On the other hand, RFCS1 and RFCS2 could have evolved independently in the Halobacteria and Methanomicrobia—a hypothesis that we do not have enough phylogenetic resolution to affirm or reject. However, data from gene context of RFCS1, shown in Figure S4, is consistent with the phylogeny. (For a more general study of gene context of archaeal DNA replication proteins, we refer the interested reader to Ref. [30]). Also, RFCS1-RFCL complexes have been shown to have some functional activity, further lending plausibility to the notion of independent gene losses [29].

Note that the long branch of RFCS2 corresponds to a change of function. Unlike RFCS and RFCS1, RFCS2 is unable to further extend the small subunit chain since it contains only one RFCS-RFCS binding site [29]. Thus, very conserved amino acid positions in RFCS and RFCS1 corresponding to the second RFCS-RFCS binding site have been allowed to drift in RFCS2 [29], resulting in the long RFCS2 branch seen in Figure 4. Also note that the RFCL rooting of the RFCS tree places the root within the eukaryotes, but is not in significant disagreement with the more sensible rooting between Archaea and eukaryotes (Figure S2).

The results for RFCS are consistent with a simpler ancestral RFC complex containing RFCL and four identical RFCS subunits. In the Archaea, we see subsequent multiple independent duplications and divergences of the distinct subunit types in both crenarchaeotes and Euryarchaea. In eukaryotes, we do not see any intermediate forms with fewer than four distinct RFCS types.

### Minichromosome Maintenance Complex

MCM complex plays a role in replication licensing [31] and DNA duplex unwinding [32]. The MCM complex consists of six homologous subunits arranged in a hexameric ring (Figure 1c). The six MCM subunits are drawn from 1, 2, 3, 4, 6, or 8 distinct sequence types, depending on phylogenetic lineage (Table 1).

**Figure 5:**
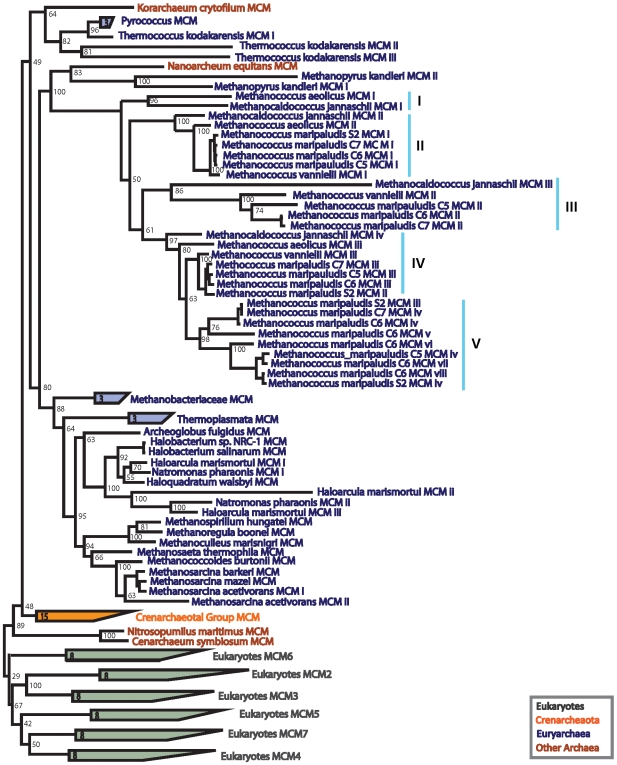
MCM phylogeny, rooted between the Archaea and the Eukaryota. The Methanococci MCM sequences show abundant gene duplication and divergence. They have been labeled I, II, III, IV, and V according to the phylogeny.

The phylogeny of the MCM subunits is shown in Figure 5 (shown uncondensed in Figure S3). As in the case of PCNA and RFCS, this phylogeny also shows general agreement with the NCBI taxonomy of the corresponding organisms. The eukaryotes, crenarchaeotes, and Euryarchaea form separate groups. Once again the basal position of *Nitrosopumilus* and *Cenarcheaum* is consistent with a distinct phylum level group, the proposed Thaumarchaeota [24]. Also as in Figures 2 and 4, the Korarchaea and Nanoarchaea sequences group with those of the Euryarchaea. Once again, given the general agreement between gene and organismal relationships, HGT between distantly related organisms does not appear in the phylogeny of the MCM subunits.

The phylogeny of the MCM subunits shows that MCM with six distinct sequence types seems to have been present in a common eukaryotic ancestor, a result previously noted by Liu et al. [33]. By contrast, the archaeal genomes vary in the number of distinct MCM sequence types they contain. The crenarchaeotes appear to contain only a single distinct MCM subunit. On the other hand, the euryarchaeotal genomes contain up to eight distinct MCM subunit genes.

The largest number of MCM genes can be found in the Methanococci. The Methanococci subunits in Figure 5 are labeled based on their phylogeny. The branch lengths between the labeled groups appear indicative of distinct roles among the subunits. The organismal members of each group vary—an indication of gene gains and losses in the Methanococci. For instance, *Methanococcus aeolicus* appears to have lost MCM III while *Methanococcus maripaludis* C6 has five MCM V sequences.

There are multiple eukaryotic MCM complexes. At least two different complexes are known to play a role in unwinding dsDNA [34], MCM2-7 [35] and MCM467 [32], [36]. MCM2467 and MCM35 complexes have also been observed [37]. In Archaea, MCM has mostly been characterized in single MCM containing organisms, and several of these MCM proteins have been shown to function as homohexamers [38]–[44]. It is worth noting, however, that MCM in *Pyrococcus furiosus* requires the presence of accessory protein GINS for unwinding DNA activity [43]. Recently it has been demonstrated that coexpression of the four MCM homologs in *Methanococcus maripaludis* S2 result in the formation of a heterohexameric complex [45]. Since *M. maripaludis* has a very robust genetic system, we anticipate that subsequent studies will reveal the need for multiple MCM homologs in this archaeon, instead of the usual single homolog in most archaea.

These results are consistent with an ancestral homohexameric MCM complex. In the Archaea, we see subsequent multiple independent duplications and divergences of the distinct subunit types in the Euryarchaea. The crenarchaeotes, on the other hand, retain the simpler ancestral configuration. In eukaryotes, we do not see any intermediate forms with fewer then six distinct sequence types implying a common eukaryotic ancestor containing six distinct MCM subunits.

## Discussion

The different numbers of distinct but homologous subunits utilized in the formation of these three complexes in different taxa represent different levels of refinement in the structure and interactions of the complexes. Complexes made up of identical subunits retain the least possibilities for refinement and specialization, while complexes made up entirely of distinct subunits hold the most possibilities for refinement and specialized interactions of each subunit. For example, the eukaryotic RFCS subunits have been shown to play a role in cell cycle regulation, serving as sensors for important processes such as cell cycle arrest and DNA damage repair [46]–[48]. Likewise, the eukaryotic MCM helicase has been shown to serve as a regulatory target in cell cycle regulation [48]. From the robust genetic system in *M. maripaludis*, we anticipate that subsequent studies will reveal the need for multiple MCM homologs in this archaeon, instead of the usual single homolog in most archaea. Similarly specialized roles have yet to be identified in the archaeal analogs of these proteins, but hints of additional function exist. Crenarchaeota exhibit differences in the PCNA interacting protein (PIP) box of proteins such as FEN1 and DNA polymerase B1-differences that are not found in the exclusively homotrimeric PCNA-containing eukaryotes, Euryarchaeota, *Cenarchaeum*, and *Nitrosopumilus* [49]. Thus, while PIP-box containing proteins in the euryarchaeota and the eukaryotes may be able to bind any of the three binding sites in the homotrimeric PCNA, PCNA interacting proteins in the crenarchaeota are known to have preferred interaction partners [21]. This suggests that functional differences may exist between homo- and heterotrimeric PCNA. We can surmise that the level of refinement of the crenarchaeotal PCNA as well as eukaryotic RFC and MCM may play a role in providing additional functionality. If true, we would expect the archaeal subunits from less refined complexes to have lesser roles than those from more refined complexes.

The archaeal branch always begins with complexes formed from exactly one PCNA, RFCS, or MCM distinct subunit type. Thereafter, the archaeal subunits duplicate and diverge, resulting in complexes with a greater level of refinement. In other words, the number of distinct subunits is always *increasing*. These refinements sometimes occur independently in multiple archaeal lineages with no evidence for HGT of distinct subunit types between different species. The agreement among our phylogenies and the concurance with other results supports the conclusions of Brochier et al. [50] that organismal phylogenies can be reconstructed from protein coding genes. It is particularly noteworthy that in all three phylogenies we discuss, the *Nitrosopumilus* and *Cenarcheaum* data are consistent with the proposal for an additional archaeal phylum, the Thaumarchaeota [24].

On the other hand, eukaryotes exhibit no changes in the number of distinct subunits. Instead, the level of refinement remains that of an ancestral Eukaryote from which the modern eukaryotes derive. In two of the cases, RFC and MCM, the ancestral eukaryotic complexes contained the maximum number of possible distinct subunits. In the other case, PCNA, the ancestral eukaryotic complex was made from three identical copies of a single distinct subunit. The same level of refinement has been retained in all modern eukaryotes surveyed in the literature [33], [51], [52] and during the course of this work.

When the number of distinct subunits increases, the duplication is followed by an initially faster evolution. This can be seen from the longer branch lengths that lead into some subunit clades, for example, the long branches of RFCS2 in Figure 4 or the long branches leading up to PCNA C1, C2, and C3 in Figure 2. This is consistent with a change in the selection on these subunits, i.e., positive selection for a different functional role [53].

Similar patterns of early complexity increase (subunit differentiation) in the common ancestral line of eukaryotes, followed by relatively stable conservation of the composition throughout subsequent speciation has been previously observed in other complexes including the 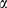 and 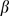 subunits of the proteasome [54] and the core histone subunits [55]. In other words, when the eukaryotic subunits are specialized, intermediate forms are often lacking. We therefore cannot be certain how the eukaryotic complexity arose in these cases. However, we can state with certainty that the many distinct archaeal subunits in the three present cases do not derive from reductive evolution of the eukaryotic complexes, as their subunit proliferation is phylogenetically independent.

Finally, it is interesting to consider the role of DNA processivity within the larger scheme of evolution in early life. Processivity was likely a requirement for the replication of large chromosomes on competitive timescales. One consequence of increased processivity in DNA replication would be the ability to retain additional copies of genes that could then potentially specialize and form more refined complexes. Ironically, the initial evolution of these three complexes may have provided themselves with the means necessary for their own subsequent refinements.

## Materials and Methods

Sequences were collected from the NCBI database and identified using BLAST [56] by their similarity to proteins identified experimentally [21]–[23], [26]–[28], [34], [35], [57]–[60]. Sequences used in this study are listed in Table S1. Multiple alignments were based on MUSCLE [61] and edited by hand using Jalview [62], and are available upon request. Columns that were judged to be poorly resolved or lacking in information content were removed prior to the maximum likelihood phylogeny. The maximum likelihood phylogeny was performed by RAxML [63] using command line arguments of the form:

./raxmlHPC-PTHREADS -T 8 -f a -x 57843 -p 83755 -N 10000 -m PROTMIXDAYHOFF

 -s alignment_file.phy

The trees presented in the main article were condensed in ARB [64]. Bootstrap values were calculated using PhyML 3.0 (http://www.atgx-montpellier.fr/phyml/) the RAxML-generated trees with their corresponding multiple alignments as the initial input [65].

## Supporting Information

Figure S1 Uncondensed PCNA phylogeny.
(0.03 MB TIF)

Figure S2 Uncondensed RFCS phylogeny, rooted by RFCL.
(0.03 MB TIF)

Figure S3 Uncondensed MCM phylogeny.
(0.04 MB TIF)

Figure S4 Genome context for the Methanomicrobiales, Methanosarcinales, Methanosaeta thermophila, and uncultured archaeon RC-I. The key shows the genes that are conserved across contexts. Uncolored genes denote that there was no homolog among these seven contexts.
(0.27 MB TIF)

Table S1 List of sequences used in this study.
(0.10 MB PDF)
