# Supplementary figures and images for: Evolution of DNA Replication Protein Complexes in Eukaryotes and Archaea
Source: PLoS One. 2010 Jun 2;5(6):e10866. doi: 10.1371/journal.pone.0010866 (PMC2880001; doi:10.1371/journal.pone.0010866)

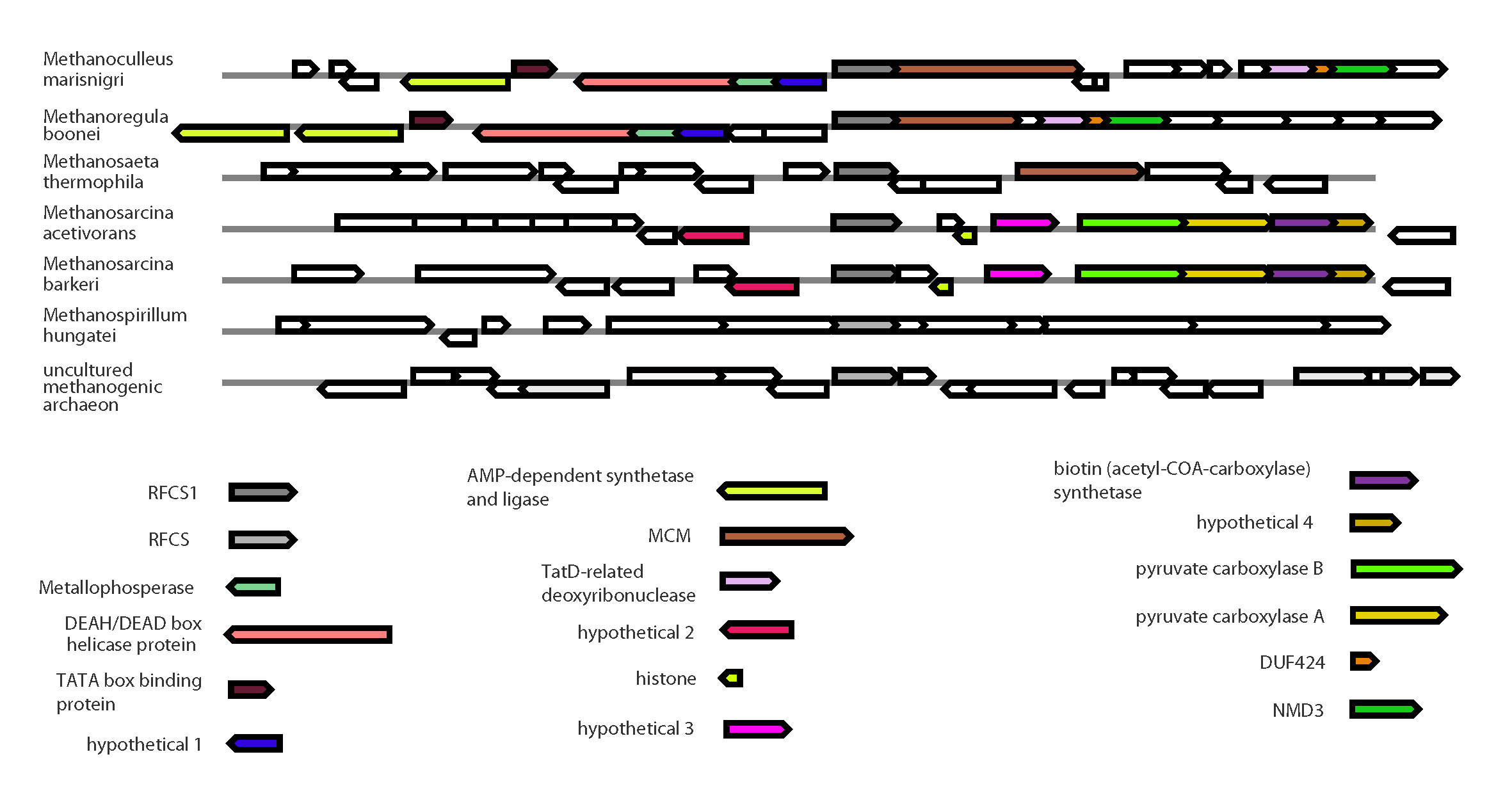

Supplement: Figure S4 — Genome context for the Methanomicrobiales, Methanosarcinales, Methanosaeta thermophila, and uncultured archaeon RC-I. The key shows the genes that are conserved across contexts. Uncolored genes denote that there was no homolog among these seven contexts. (0.27 MB TIF) [file pone.0010866.s004.tif]
