# Supplementary material for: Evolution of DNA Replication Protein Complexes in Eukaryotes and Archaea
Source: PLoS One. 2010 Jun 2;5(6):e10866. doi: 10.1371/journal.pone.0010866 (PMC2880001; doi:10.1371/journal.pone.0010866)
Supplement: Table S1 — List of sequences used in this study. (0.10 MB PDF) [file pone.0010866.s005.pdf]

Organism	PCNA		RFCS		MCM	
	Name	GI number	Name	GI number	Name	GI number
**Archaea**						
**Crenarchaeota**						
Aeropyrum pernix K1	PCNA C1<br>PCNA C2<br>PCNA C3	gi|118431047<br>gi|14601898<br>gi|14600497	RFCS	gi|118431491	MCM	gi|118430924
Caldivirga maquilingensis IC-167	PCNA C1<br>PCNA C2	gi|159041941<br>gi|159040634	RFCS i<br>RFCS ii<br>RFCL	gi|159041490<br>gi|159041324<br>gi|159041491	MCM	gi|159040966
Cenarchaeum symbiosum A	PCNA	gi|118576605	RFCS	gi|118576898	MCM	gi|118576896
Hyperthermus butylicus DSM 5456	PCNA C1<br>PCNA C2<br>PCNA C3	gi|124028484<br>gi|124028141<br>gi|124026921			MCM	gi|124027777
Ignicoccus hospitalis KIN4/I	PCNA C1<br>PCNA C2<br>PCNA C3	gi|156937647<br>gi|156937506<br>gi|156937403	RFCS<br>RFCL	gi|156938086<br>gi|156938087	MCM	gi|156938084
Metallosphaera sedula DSM 5348	PCNA C1<br>PCNA C2<br>PCNA C3	gi|146304555<br>gi|146302836<br>gi|146304996	RFCS	gi|146304798	MCM	gi|146304791
Nitrosopumilus maritimus SCM1	PCNA	gi|161529263	RFCS	gi|161527748	MCM	gi|161527750
Pyrobaculum aerophilum str. IM2	PCNA C2<br>PCNA C1	gi|18312128<br>gi|18313780	RFCS ii<br>RFCS i	gi|18312140<br>gi|18312778	MCM	gi|18312259
Pyrobaculum arsenaticum DSM 13514	PCNA C2<br>PCNA C1	gi|145592555<br>gi|145591989	RFCS i<br>RFCS ii	gi|145592047<br>gi|145590269	MCM	gi|145590378
Pyrobaculum calidifontis JCM 11548	PCNA C2<br>PCNA C1	gi|126460734<br>gi|126459603	RFCS ii<br>RFCS i	gi|126459198<br>gi|126458633	MCM	gi|126458712
Pyrobaculum islandicum DSM 4184	PCNA C2<br>PCNA C1	gi|119872181<br>gi|119871952	RFCS ii<br>RFCS i	gi|119873156<br>gi|119872170	MCM	gi|119872659
Thermofilum pendens Hrk 5	PCNA	gi|119719536	RFCS	gi|119719185	MCM	gi|119719412
Thermoproteus neutrophilus V24Sta	PCNA C1<br>PCNA C2	gi|171186131<br>gi|171186437	RFCS ii<br>RFCS i	gi|171185161<br>gi|171186449	MCM	gi|171184589
Staphylothermus marinus F1	PCNA C1<br>PCNA C2<br>PCNA C3	gi|126465543<br>gi|126465899<br>gi|126466255	RFCS	gi|126466118	MCM	gi|126466120
Sulfolobus acidocaldarius DSM 639	PCNA C1<br>PCNA C2<br>PCNA C3	gi|70607045<br>gi|70606607<br>gi|70606615	RFCS	gi|70606693	MCM	gi|70606686
Sulfolobus solfataricus P2	PCNA C1<br>PCNA C2<br>PCNA C3	gi|13814221*<br>gi|15897331<br>gi|15897339	RFCS	gi|15897670	MCM	gi|15897676
Sulfolobus tokodaii str. 7	PCNA C1<br>PCNA C2<br>PCNA C3	gi|15921180<br>gi|15920600<br>gi|15920590	RFCS	gi|15920690	MCM	gi|15920683
**Euryarchaeota**						
Archaeoglobus fulgidus DSM 4304	PCNA	gi|11497947	RFCS	gi|11499642	MCM	gi|11498128
Haloarcula marismortui ATCC 43049	PCNA	gi|55379458	RFCS1<br>RFCS2	gi|55379214<br>gi|55379148	MCM iii<br>MCM i<br>MCM ii	gi|55378998<br>gi|55379381<br>gi|55376226
Halobacterium salinarum R1	PCNA	gi|169094648			MCM	gi|169236761
Halobacterium sp. NRC-1	PCNA	gi|15791072	RFCS1<br>RFCS2	gi|15791090<br>gi|15791058	MCM	gi|15791012
Haloquadratum walsbyi DSM 16790	PCNA	gi|110669536	RFCS1<br>RFCS2	gi|110669521<br>gi|110669279	MCM	gi|110669512
Methanobrevibacter smithii ATCC 35061	PCNA	gi|148643197	RFCS	gi|148643236	MCM	gi|148642570
Methanocaldococcus jannaschii DSM 2661	PCNA	gi|15668422	RFCS	gi|15669613	MCM i<br>MCM ii<br>MCM iv<br>MCM iii	gi|15668539<br>gi|15669151<br>gi|15669682<br>gi|10954504
Methanococcoides burtonii DSM 6242	PCNA	gi|91774116			MCM	gi|91774341
Methanococcus aeolicus Nankai-3	PCNA	gi|150401128	RFCS<br>RFCL	gi|150401734<br>gi|150401783	MCM iii<br>MCM ii<br>MCM i	gi|150401567<br>gi|150400922<br>gi|150401648
Methanococcus maripaludis C5	PCNA	gi|134046721	RFCS	gi|134046237	MCM i	gi|134045613

					MCM ii<br>MCM iv<br>MCM iii	gi|134045684<br>gi|134046149<br>gi|134046676
Methanococcus maripaludis C6	PCNA	gi|159905345	RFCS	gi|159904872	MCM iv<br>MCM v<br>MCM vii<br>MCM vi<br>MCM viii<br>MCM iii<br>MCM i<br>MCM ii	gi|159904426<br>gi|159904502<br>gi|159904526<br>gi|159904809<br>gi|159904826<br>gi|159905304<br>gi|159906017<br>gi|159906217
Methanococcus maripaludis C7	PCNA	gi|150402910	RFCS	gi|150403344	MCM iii<br>MCM i<br>MCM iv<br>MCM ii	gi|150402951<br>gi|150402193<br>gi|150401955<br>gi|150401992
Methanococcus maripaludis S2	PCNA	gi|45359274	RFCS	gi|45357990	MCM ii<br>MCM iv<br>MCM iii<br>MCM i	gi|45357593<br>gi|45358033<br>gi|45358311<br>gi|45358587
Methanococcus vannielii SB	PCNA	gi|150399761	RFCS	gi|150400153	MCM iii<br>MCM i<br>MCM ii	gi|150399775<br>gi|150399102<br>gi|150399021
Methanoculleus marisnigri JR1	PCNA	gi|126179409	RFCS1<br>RFCS2	gi|126179196<br>gi|126179181	MCM	gi|126179195
Methanopyrus kandleri AV19	PCNA	gi|20094466	RFCS	gi|20093446	MCM i<br>MCM ii	gi|20094401<br>gi|20094556
Methanoregula boonei 6A8	PCNA	gi|154150935	RFCS1<br>RFCS2	gi|154151216<br>gi|154151206	MCM	gi|154151215
Methanosaeta thermophila PT	PCNA	gi|116754334	RFCS	gi|116753581	MCM	gi|116753578
Methanosarcina acetivorans C2A	PCNA	gi|20089009	RFCS1<br>RFCS2	gi|20089555<br>gi|20089039	MCM i<br>MCM ii	gi|20089566<br>gi|20092625
Methanosarcina barkeri str. Fusaro	PCNA	gi|73668418	RFCS1<br>RFCS2	gi|73669094<br>gi|73668390	MCM	gi|73669105
Methanosarcina mazei Go1	PCNA	gi|21227499	RFCS1<br>RFCS2	gi|21227923<br>gi|21227528	MCM	gi|21227938
Methanosphaera stadtmanae DSM 3091	PCNA	gi|84490309	RFCS	gi|84489225	MCM	gi|84489176
Methanospirillum hungatei JF-1	PCNA	gi|88602763	RFCS1<br>RFCS2	gi|88602285<br>gi|88602126	MCM	gi|88602276
Methanothermobacter thermautotrophicus str. Delta H	PCNA	gi|15679312	RFCS	gi|15678269	MCM	gi|15679758
Natronomonas pharaonis DSM 2160	PCNA	gi|76800929	RFCS1<br>RFCS2	gi|76801102<br>gi|76801186	MCM i<br>MCM ii	gi|76803181<br>gi|76802579
Picrophilus torridus DSM 9790	PCNA	gi|48478388	RFCS	gi|48477659	MCM	gi|48478289
Pyrococcus abyssi GE5	PCNA	gi|14521578	RFCS<br>RFCL	gi|14520328<br>gi|14520329	MCM	gi|14521639
Pyrococcus furiosus DSM 3638	PCNA	gi|18977355	RFCS	gi|18976465	MCM	gi|18976854
Pyrococcus horikoshii OT3	PCNA	gi|14590549	RFCS	gi|14590058	MCM	gi|14590502
Thermococcus kodakarensis KOD1	PCNA ii<br>PCNA i	gi|57640517<br>gi|57640470	RFCS	gi|57642153|	MCM iii<br>MCM ii<br>MCM i	gi|57640031<br>gi|57641296<br>gi|57641555
Thermoplasma acidophilum DSM 1728	PCNA	gi|16081965	RFCS	gi|16082630	MCM	gi|16081861
Thermoplasma volcanium GSS1	PCNA	gi|14325326	RFCS	gi|14325757	MCM	gi|13541863
uncultured methanogenic archaeon RC-I			RFCS	gi|147918695		
**Korarchaeota**						
Korarchaeum cryptofilum OPF8	PCNA	gi|170291142	RFCS	gi|170290625	MCM	gi|170290965
**Nanoarchaeota**						
Nanoarchaeum Equitans	PCNA	gi|41615320	RFCS	gi|41614964	MCM	gi|41615073
**Eukaryotes**						
Arabidopsis thaliana	PCNA ii<br>PCNA i	gi|15227564<br>gi|15222379	RFCS5<br>RFCS3<br>RFCS2<br>RFCS4	gi|15241031<br>gi|15223967<br>gi|18395021<br>gi|15221697	MCM2<br>MCM3<br>MCM4<br>MCM5<br>MCM6<br>MCM7	gi|145336465<br>gi|3036819<br>gi|4544386<br>gi|15226146<br>gi|22327575<br>gi|675491

<table>
<tr><td>Aspergillus fumigatus Af293</td><td>PCNA gi|70990780</td><td></td><td>MCM2 gi|70999003
MCM3 gi|146322416
MCM4 gi|71001116
MCM5 gi|70985168
MCM6 gi|146324155
MCM7 gi|71001332</td></tr>
<tr><td>Caenorhabditis elegans</td><td>PCNA gi|71995072</td><td></td><td>MCM2 gi|71997752
MCM3 gi|17562700
MCM4 gi|17508417
MCM5 gi|17554306
MCM6 gi|71997563
MCM7 gi|17562702</td></tr>
<tr><td>Ciona intestinalis</td><td>PCNA gi|198433566</td><td></td><td></td></tr>
<tr><td>Dictyostelium discoideum AX4</td><td>PCNA gi|66806789</td><td>RFCS2 gi|66802182
RFCS3 gi|66800141
RFCS4 gi|66808355
RFCS5 gi|66812244</td><td></td></tr>
<tr><td>Drosophila melanogaster</td><td></td><td></td><td>MCM2 gi|17137132
MCM3 gi|24639835
MCM4 gi|60677941
MCM5 gi|24645774
MCM6 gi|17530827
MCM7 gi|17647617</td></tr>
<tr><td>Drosophila simulans</td><td>PCNA ii gi|195585035
PCNA i gi|195580243</td><td></td><td></td></tr>
<tr><td>Homo sapiens</td><td>PCNA gi|4505641</td><td>RFCS2 gi|31563534
RFCS3 gi|54696204
RFCS4 gi|4506491
RFCS5 gi|47115239
RFCL gi|32528306</td><td>MCM2 gi|33356547
MCM3 gi|6631095
MCM4 gi|33469917
MCM5 gi|23510448
MCM6 gi|7427519
MCM7 gi|1255617</td></tr>
<tr><td>Oryza sativa</td><td>PCNA gi|115449323</td><td></td><td></td></tr>
<tr><td>Plasmodium falciparum 3D7</td><td></td><td></td><td>MCM2 gi|124808572
MCM3 gi|124506457
MCM4 gi|124513100
MCM5 gi|124805740
MCM6 gi|124513814
MCM7 gi|124511708</td></tr>
<tr><td>Saccharomyces cerevisiae</td><td>PCNA gi|6319564</td><td>RFCS2 gi|6322528
RFCS3 gi|151944257
RFCS4 gi|6324478
RFCS5 gi|6319562
RFCL gi|6324791</td><td>MCM2 gi|3912
MCM3 gi|6320803
MCM4 gi|6325276
MCM5 gi|6323304
MCM6 gi|41629691
MCM7 gi|6319679</td></tr>
<tr><td>Trypanosoma brucei TREU927</td><td></td><td></td><td>MCM2 gi|71755893
MCM3 gi|84043638
MCM4 gi|74025442
MCM5 gi|71755435
MCM6 gi|74025322
MCM7 gi|74026210</td></tr>
</table>

*amino acid sequence obtained from translated nucleotides
